# Evaluation of a Redesigned Personal Protective Equipment Gown

**DOI:** 10.1093/cid/ciz520

**Published:** 2019-09-13

**Authors:** Frank A Drews, Diane Mulvey, Kristina Stratford, Matthew H Samore, Jeanmarie Mayer

**Affiliations:** 1 Department of Psychology, Department of Internal Medicine, University of Utah, Salt Lake City; 2 Division of Epidemiology, Department of Internal Medicine, University of Utah, Salt Lake City

**Keywords:** PPE, evaluation, gown, usability

## Abstract

**Background:**

In healthcare, the goal of personal protective equipment (PPE) is to protect healthcare personnel (HCP) and patients from body fluids and infectious organisms via contact, droplet, or airborne transmission. The critical importance of using PPE properly is highlighted by 2 potentially fatal viral infections, severe acute respiratory syndrome–associated coronavirus and Ebola virus, where HCP became infected while caring for patients due to errors in the use of PPE. However, PPE in dealing with less dangerous, but highly infectious organisms is important as well. This work proposes a framework to test and evaluate PPE with a focus on gown design.

**Methods:**

An observational study identified issues with potential for contamination related to gown use. After redesigning the existing gown, a high-fidelity patient simulator study with 40 HCP as participants evaluated the gown redesign using 2 commonly performed tasks. Variables of interest were nonadherence to procedural standards, use problems with the gown during task performance, and usability and cognitive task load ratings of the standard and redesigned gowns.

**Results:**

While no differences were found in terms of nonadherence and use problems between the current and the redesigned gown, differences in usability and task load ratings suggested that the redesigned gown is perceived more favorably by HCP.

**Conclusions:**

This work proposes a framework to guide the evaluation of PPE. The results suggest that the current design of the PPE gown can be improved in usability and user satisfaction. Although our data did not find an increase in adherence to protocol when using the redesigned gown, it is likely that higher usability and lower task load could result in higher adherence over longer periods of use.

## Teaser

To improve personal protective equipment adherence, a simulator study evaluated a redesigned gown for usability in comparison to a standard gown. The results of this study suggest that the redesigned gown was more usable and reduced cognitive task load.

Personal protective equipment (PPE) is used in a wide range of industries to protect workers from exposure to workplace hazards and is designed to address requirements specific to the context of its use. In healthcare, the goal of PPE is to protect healthcare personnel (HCP) from body fluids and infectious organisms via contact, droplet, or airborne transmission. The critical importance of using PPE properly is highlighted by 2 potentially fatal viral infections: severe acute respiratory syndrome–associated coronavirus (SARS-CoV) and Ebola virus. The SARS-CoV epidemic in Asia and Toronto during 2002–2003 led to >8000 cases, with most transmission occurring in healthcare settings, and the more recent Ebola outbreak in West Africa during 2014–2016 resulted in >28 000 infected individuals with 11 000 fatalities [1]. In both epidemics, HCP became infected while caring for patients due to errors in the use of PPE, raising the question of how well PPE is designed to support HCP [2].

One perspective of proper PPE use focuses on equipment design and assumes successful use is assured if HCP receive adequate training. This position is implicit in the hazard-barrier-target model and the accident evolution and barrier function model and implies that PPE can be designed and evaluated in isolation. An alternative approach is to conceptualize safe and effective PPE use from a sociotechnical perspective. Factors to consider that may interact and influence PPE use include the equipment, user, task, and the environment. Equipment designed for HCP providing routine care may result in failures when HCP practice in emergency settings. For example, HCP who rush into a patient’s room while distracted by an alarm may not don PPE properly. Cavazza and colleagues demonstrated that organizational and psychosocial factors influence risk behavior and attitudes of individuals toward PPE [3]. In addition, the organizational safety climate affects employees’ shared perceptions of safety policies, procedures, and practices as well as potential for unsafe behaviors [4].

PPE use in healthcare involves 3 phases: (1) donning, (2) while providing patient care, and (3) doffing. Issues or errors during any of these phases can lead to a risk of contamination to the HCP. Incorrect technique or sequence in donning can expose HCP during patient care, or sets HCP up for a doffing failure. Contamination of HCP can occur during patient care if PPE is damaged, has design flaws, or if HCP circumvent protection (ie, reaching under PPE). Risks of contamination during doffing can be due to an incorrect removal technique, improper handling and disposal of PPE, or by damaging PPE to expose HCP.

One study using a simulation to assess use of PPE during donning, doffing, and while HCP performed care tasks used observation [5]. Each of the 10 participants committed at least 1 PPE breach, and breaches occurred during all use phases. Donning issues included failures in properly tying the gown; touching unprotected body areas with contaminated PPE during care was common; and errors in removing PPE included doffing sequence deviations and improper mask removal. A qualitative study with 325 observations of isolation rooms in multiple hospitals was conducted to identify and characterize PPE use failures [6]. Two hundred eighty-three observed failures were categorized as violations (n = 102), process/procedural mistakes (n = 144), and slips (n = 37). Examples for these failures were entering the room without required PPE (violation), PPE doffing sequence errors (mistake), and wiping one’s face with potentially contaminated gowned forearms (slip). The authors concluded that given the range of contributors to self-contamination events, no single strategy is sufficient to reduce transmission risk. Relatively little work has focused on redesigning PPE to make it more user friendly and to promote proper use. One example of a study that did use a human factors–based approach developed a standardized PPE storage cart with picture labels and PPE use instructions [7]. Baseline PPE compliance was only 47%, but increased to 81% after cart introduction.

Taken together, HCP remain at risk of exposure to infectious agents due to improper use of PPE. There are significant challenges in reducing failures in PPE use given the complex nature of the equipment, the donning and doffing procedures, the clinical tasks that affect the ability of PPE to protect HCP, and how PPE affects HCP in performing clinical tasks. The goal of this study was to provide a framework for the evaluation of PPE, and to apply this framework to develop and evaluate a redesigned gown prototype, intended to address the issues observed with a standard isolation gown.

## BACKGROUND WORK

### Observation of Gown Donning and Doffing

We previously conducted an observational study to examine the potential for HCP in clinical practice to self-contaminate during donning and doffing of PPE [8]. To summarize, 48 observations of randomly selected HCP were performed at a university-based medical center with the hospital PPE guidelines based on Centers for Disease Control and Prevention recommendations used as the expectation for proper gown use. Only 42% of HCP appropriately tied their gowns in the back, and subsequently removed the gowns by breaking the tie. Forty-eight percent potentially self-contaminated while doffing, and 42% did not follow the hospital-specified technique for gown disposal. The observations indicated that a large number of participants were at risk of contamination and suggested that there was a need to identify gown redesign opportunities. We determined that critical gaps in the standard gown included errors in closing the gown, potential for HCP exposure while providing care due to movement of the light gown material, difficulties with easily removing, and touching the external contaminated surfaces while doffing.

## METHODS

This study used an iterative design approach to develop a prototype standard isolation gown, and then evaluated use of the standard and redesigned gowns under simulated conditions. Approval for this study was obtained by the University of Utah Institutional Review Board.

### Development of a Prototype Redesigned Gown

We considered how to address the errors that we observed HCP made in how they used the standard gown that could have placed them at risk of contamination. Iterative changes were made to the standard gown, piloted, and then discussed by 4 members of the research team until a final prototype was developed (D. M., F. A. D., K. S., and J. M.).

### Evaluation of the Redesigned Gown

The usability and effectiveness of the redesigned gown was evaluated in comparison to the standard gown. The hypotheses were as follows: (1) the redesigned gown reduces the likelihood of procedural nonadherence during clinical tasks and doffing; (2) usability ratings for the redesigned gown are higher; and (3) the task load for using the redesigned gown is lower.

#### Simulation Center

The study took place in the Simulation Learning Center at the University of Utah, which consists of a centralized control room and fully functional simulated critical care suites, each suite with a high-fidelity human patient simulator (SimMan 3G). Data collection involved audio and video recording of participants for later analysis.

#### Participants

Study staff recruited nurses (n = 20) and nurse aides (n = 20) from the University of Utah Hospital to participate. Inclusion criteria included current employment on a clinical unit as a nurse/nurse aide and with experience in PPE use. Participation was voluntary.

#### Study Design

A 2 (task) × 2 (gown) nested, repeated measurements design was used to evaluate the redesigned gown before, during, and after completing standardized clinical tasks. The order of gown use and scenario was counterbalanced across participants.

#### Measures

Behavioral and subjective measures were used to assess the effectiveness and usability of the redesigned gown. The behavioral measures focused on nonadherence to appropriate use of PPE from video recordings of participants completing each scenario. The recordings captured HCP donning, use and function of PPE while performing standardized clinical tasks, and gown doffing. A schema to code video recordings was developed with the following categories: donning gown (if and how gown was closed), PPE-related issues (exposure while squatting, tie or gown touches floor), doffing gown (pulling gown from waist, balling up gown). Coding classified behaviors as adherent or nonadherent per hospital policy. Standardized instructional materials were used to train 3 coders to identify nonadherence in the recordings. Once performance of the coders met criterion of 95% between-coder agreement in a set of training videos, they coded the study data.

Subjective measures assessed participants’ perceptions of the redesigned gown. Workload and usability were measured using the NASA Task Load Index (NASA-TLX) and the System Usability Scale (SUS) [9, 10]. The NASA-TLX is a subjective, multidimensional questionnaire used in complex sociotechnical systems domains such as aviation and healthcare to assess perceived workload on 6 dimensions including mental demand, physical demand, temporal demand, performance, effort, and frustration. The SUS is a 10-item attitude Likert scale that measures subjective usability of a system by yielding a single score on a scale of 0–100 and allows for a comparison across different systems using normative data [11]. Participants were also asked to rate gowns on attributes of ease of use during donning, clinical care, doffing, convenience, design, comfort, and risk of contamination on a Likert-type scale ranging from 1 (standard gown best), to 4 (both gowns equal), to 7 (redesigned gown best). Finally, participants provided additional verbal feedback comparing the gowns.

#### Procedure

After arrival in the simulation center, participants completed a consent form and survey to describe their professional background and experience. All participants reviewed a brief presentation and were given an opportunity to ask questions on the design, attributes, and use of the redesigned gown. They were then introduced to the simulator and given patient information along with a brief description of the task they were to perform. The simulated patients were in isolation precautions with signage posted outside the patient room indicating the required PPE. Participants performed 2 scenarios, with a different gown (standard or redesigned) made available prior to the start of each scenario. Upon completion of each of the 2 tasks, participants were given the NASA-TLX and SUS. After finishing 2 NASA-TLX and SUS questionnaires, each participant responded to the survey rating the 2 gowns and provided any additional verbal comments.

#### Scenarios

Participants performed 1 task per scenario, with the tasks being discontinuation of a peripheral intravenous saline lock or the emptying of a Foley catheter drainage bag. Table 1 lists the task steps involved.

**Table 1.** Individual, Sequential Task Steps for Both Tasks (Discontinuation of Peripheral IV Saline Lock and Emptying Foley Catheter Drainage Bag) Participants Needed to Perform.

Step	Discontinuation of a Peripheral IV Saline Lock	Emptying a Foley Catheter Drainage Bag
1	Perform hand hygiene and don gloves and gown.	Perform hand hygiene, don gloves and gown, and get graduate container.
2	Assess site for complications (ie, infection, infiltration or phlebitis).	Check catheter tubing for kinks and verify patient not lying on it.
3	Place sterile gauze above site and withdraw catheter using slow steady motion. Keep hub parallel to skin.	Carefully open port drainage valve. Lift bag to drain contents into graduate container. Avoid touching port to container.
4	Apply pressure to site using sterile gauze until homeostasis achieved.	Once draining finished, close port.
5	Inspect catheter integrity after removal; note tip integrity and length.	Transport graduate container to commode.
6	Clean site.	Place graduate container on level flat surface and measure amount of urine at eye level.
7	Apply clean folded gauze dressing over insertion site and secure dressing.	Empty content of graduate container into commode.
8	Discard supplies, remove gloves, gown, and perform hand hygiene.	Discard graduate container.
9		Ensure drainage bag and tubing not touching floor. Secure bag to non-movable part of bed. Check tubing for kinks and ensure patient not lying on it.
10		Ensure patient has call light and is comfortable. Open privacy curtain, remove gloves and gown, and perform hand hygiene.

## RESULTS

### Redesigned Gown Prototype

**Figure 1.**
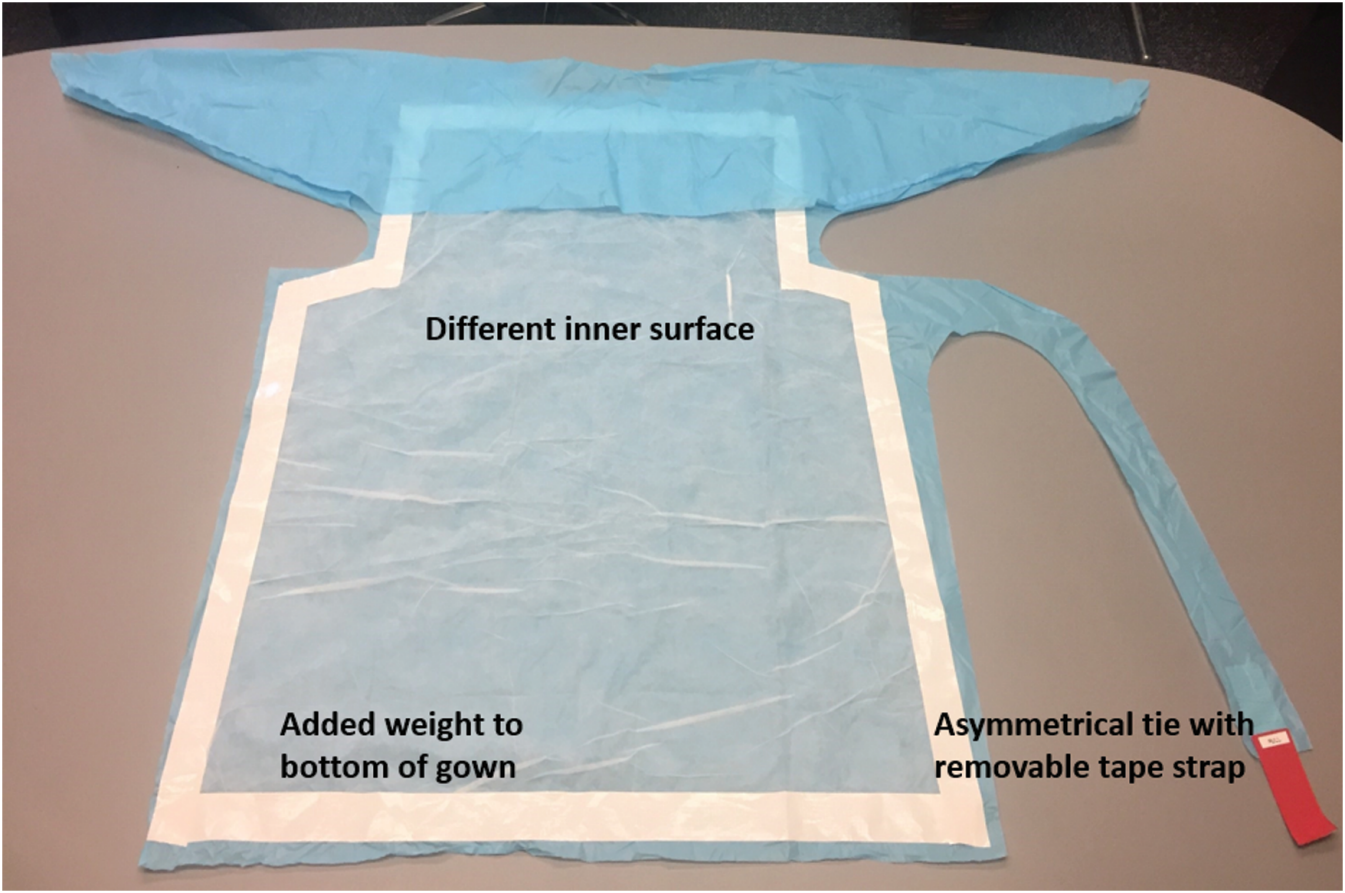
Redesigned gown with new features.

Our main gown redesign considerations focused on improving the closure mechanism, providing visual cues to demarcate the contaminated outer from the clean inner surfaces, weighing down the gown material for better coverage, and making gown removal easier by adding perforations to the tie. A closure mechanism using an asymmetrical closure approach was favored, with the gown secured by pulling a single strap from the back to front. An adhesive strip covered by red tape was placed at the end of the strap. Pulling the tape off the adhesive strip allowed for strap securement to the front of the gown. Another design change addressed the uniform color of the gown that prevented HCP from distinguishing between the clean inner and potentially contaminated outer surfaces. Initial designs ranged from a different-colored inside gown material, to applying tape to mark the clean surface. Pilot testing revealed that a combination of these approaches was most promising. As the lightweight and static-inducing gown material had a tendency to make contact with objects as well as expose the HCP lower body during movement, we determined the bottom edge of the gown needed to be heavier. To create this additional weight, the tape marking the inner surface was also attached along the bottom of the gown. Finally, we enhanced the perforated areas of the gown material to break more easily during the doffing process. Figure 1 shows the redesigned gown.

### Evaluation of Gowns

Each participant trialed both gowns, using a different gown for the 2 scenarios.

### Behavioral Measures

Analyses focused on the percentage of procedural nonadherence during donning (eg, standard gown not secured, redesigned gown strap taped in back) and doffing (eg, unties standard gown). PPE-related issues during task performance (eg, exposure of clothing under gown while squatting, gown touching floor) were analyzed using rate per gown type. Aggregated across both scenarios, donning nonadherence occurred in 35.8% with the standard gown, and 32.3% with the redesigned gown, while nonadherence during doffing occurred at 39.7% and 36.6%, respectively. Performance-related issues during task performance occurred on average 0.9 times (standard deviation [SD], .9) with the standard gown, and 1.2 times (SD, .76) with the redesigned gown. To test for differences between the 2 gown designs, statistical analyses were performed for the frequency of nonadherence and PPE-related issues during gown use, but in all cases no significant differences were found.

### Subjective Measures

**Figure 2.**
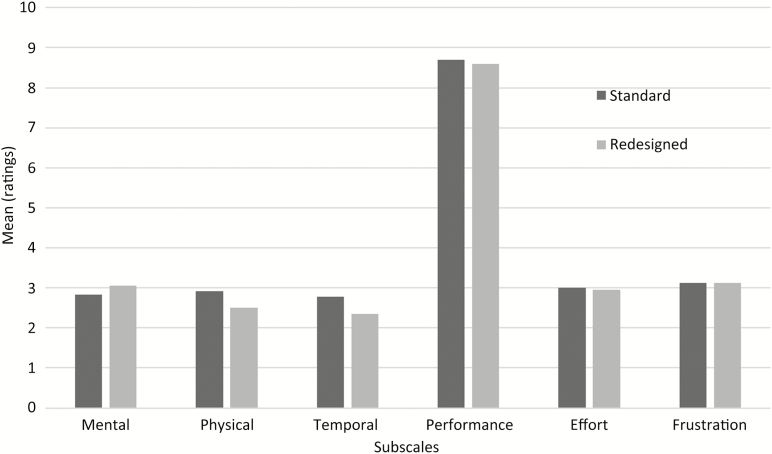
Results of the NASA Task Load Index.

**Figure 3.**
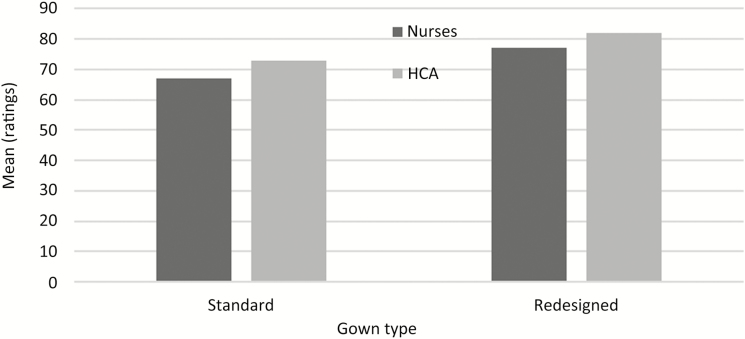
System Usability Scale scores. Abbreviation: HCA, Health Care Aids/Nurse Aids.

The NASA-TLX analyses revealed that when using the redesigned gown, participants perceived a significant reduction in temporal demand, *t*(39) = 1.75; *P* = .044. In addition, a statistical trend suggested a reduction of physical demand, *t*(39) = 1.45; *P* = .077 (Figure 2) when using the redesigned gown. None of the other subscales of the NASA-TLX (mental demand, subjective performance, subjective effort, and experienced frustration) revealed any significant differences between conditions (all *t* values <1). Analysis of the SUS using a 2 (gown) × 2 (group) design demonstrated a significant increase in satisfaction when using the redesigned gown, *F*(1, 32) = 4.49; *P* = .04 (Figure 3), without a difference between groups, *F*(1, 32) = .025; *P* = .876.

### Gown Attributes

**Figure 4.**
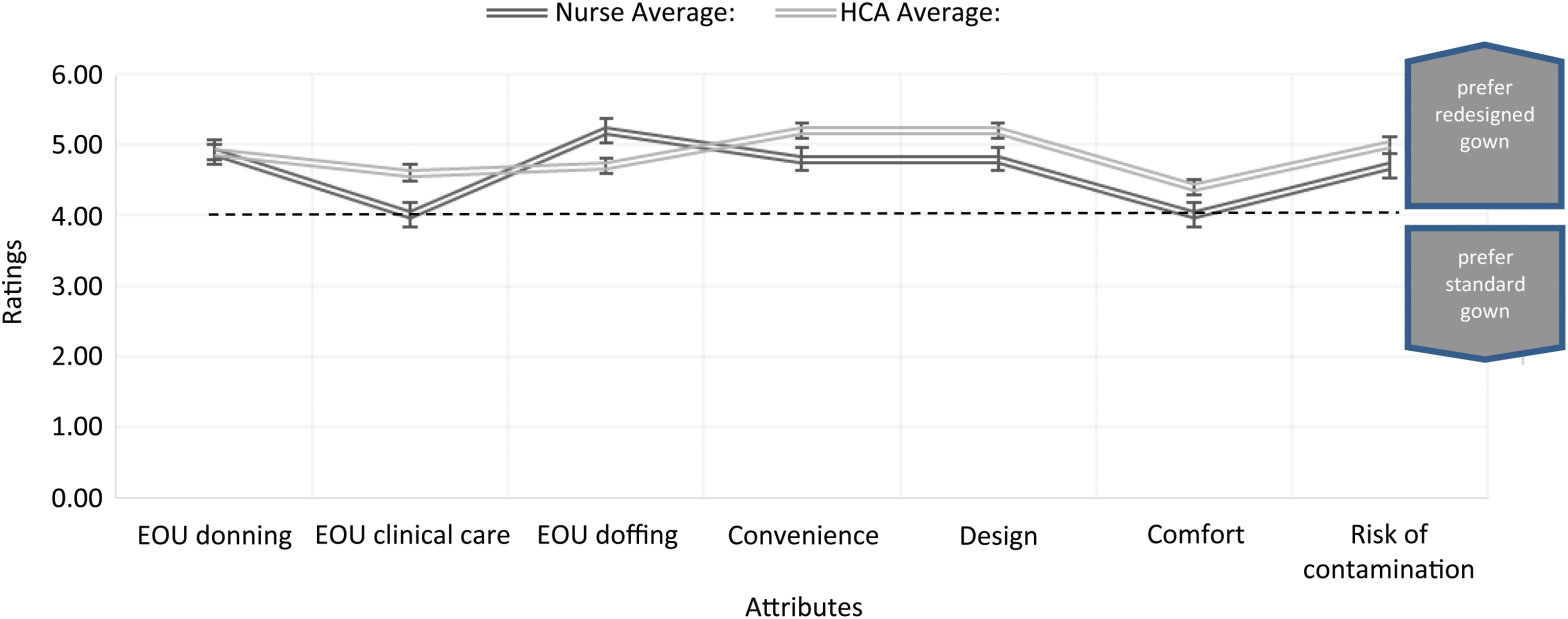
Ratings on gown attributes, by participant group. Dashed line indicates equivalence between gowns. Abbreviations: EOU, Ease of Use; HCA, Health Care Aids/Nurse Aids.

Participants evaluated the gowns directly by rating 7 gown attributes (ease of use during donning, clinical care, doffing, convenience, design, comfort, and risk of contamination) with results from both nurses and nurse aides shown in Figure 4. No statistical difference in ratings between the HCP groups was found. Ease of use during donning, clinical care, and doffing for both groups had mean ratings of 4.9, 4.33, and 4.93, respectively. Convenience, design, comfort, and risk of contamination had mean rankings of 4.95, 4.98, 4.18, and 4.8, respectively. The redesigned gown rated higher than the standard gown for all gown attributes, although the difference was smallest with regard to ease of use in clinical care and comfort.

### Participant Comments

Participants’ voluntary comments were categorized as either having positive or negative attribute valence and are listed within categories with their frequencies and percentages in Table 2. The majority of participants described as positive attributes of the redesigned gown the closure mechanism, inner/outer surface differentiation, added weight, improved fit, and ease of gown breaking during doffing. While more of the comments for the redesigned gown were positive, there were concerns including cost, fit, and weight of the gown, as well as that the gown material may lead to sweating. The ratio of positive to negative attributes was 1.4 for the redesigned gown and 0.47 for the standard gown, with positive comments clearly outweighing negative comments for the redesigned gown.

**Table 2.** Counts and Percentages (In Brackets) of Concerns and the Valence of Gown Attributes for the Standard and Redesigned Gown as Expressed by Participants at the End of the Study.

Redesigned gown			Standard gown		
**Positive**	Liner/exposure	11 (17.4%)	**Positive**	Easy use	4 (57.1)
	Easier/faster	22 (34.9)		Familiar	1 (14.3)
	Closure	14 (22.2)		Closure	2 (28.6)
	Fit/weight	16 (25.4)			
	**TOTAL**	**63 (90)**		**TOTAL**	**7 (10)**
**Negative**	Hot	10 (22.2)	**Negative**	Hot	2 (13.3)
	Fit/weight	9 (20.0)		Fit	2 (13.3)
	Liner/exposure	7 (15.5)		Exposure	1 (6.6)
	Closure	7 (15.5)		Closure	8 (53.3)
	Unfamiliar	7 (15.5)		Frustrating	2 (13.3)
	Bulky	5 (11.1)			
	**TOTAL**	**45 (75)**		**TOTAL**	**15 (25)**

## Discussion

Deviations and errors in PPE use are common and have been documented previously [12, 13]. While some authors attribute these issues as “natural flaws” [14], the present study proposes a framework in which to test and evaluate the usability and effectiveness of PPE in the simulator setting to identify and eliminate potential gown design issues. Behavior and subjective measures provide a systematic and comprehensive approach in assessing PPE. The framework was used to test the impact of a redesigned gown prototype.

While the behavior measures from observations of participants using the standard vs redesigned gown in the simulated clinical scenarios did not show differences, the assessment was biased in favor of the familiar, traditional gown over the unfamiliar prototype. However, differences were identified on the subjective measurements of the participants’ perceptions of the standard and redesigned gowns. Participants experienced less time pressure, expressed higher usability, and rated overall the redesigned gown as superior on numerous attributes. In their comments, participants described positive attributes of the redesign while expressing some concerns.

We were unable to establish a link between the gown redesign and improved adherence. However, changes in adherence are difficult to observe in general, and especially so in the relatively short time participants used the redesigned gown in this study. Nonetheless, some support for this perspective comes from work that examined the value of information technology investment in healthcare. This work suggests that the best predictor for changes in organizational performance is actual technology use, not technology investment [15]. Thus, it is plausible to assume that increased usability will affect use, with usability becoming a predictor for behavior and ultimately adherence. This relationship has been established by work demonstrating that perceived usability predicts use and behavior [16]. Thus, adherence could increase, but it will take longer observations to observe such change.

Consistent with this point, a next step in evaluating the redesigned gown would be to introduce the gown in the clinical context and perform use observations and usability assessments. This approach would potentially demonstrate the validity of the simulator study, and test the redesigned gown in a more complex, sociotechnical system.

Finally, the redesigned gown addressed some of the issues that were observed previously [8]. While the redesign may not solve all issues identified, some were addressed by the redesign. However, other improvements (eg, multiple, break-off points with strap adhesive for better adjustment of fit, improved fabric, multiple gown sizes) may lead to additional improvements.

There are some limitations associated with this study. These limitations relate to the relatively small sample size and the limited number of clinical tasks used. Also, the comparison of the 2 gown designs favored the traditional gown design since participants have been using this gown over many years on a daily basis, while never having used the redesigned gown. Another potential limitation relates to the fact that this study limited the evaluation of the redesigned gown to nurses and nurses’ aides. However, challenges and attitudes related to precaution practices and PPE use differ between different types of providers. Future work on the redesigned gown should include other healthcare workers while they perform representative tasks to assess the benefits of the gown. In addition, the utility of contact precautions my also vary upon type of provider, which requires additional, provider-specific and precaution-specific task analyses.

Overall, this work is part of a broader human factors initiative of introducing a sociotechnical perspective into infection prevention and control. Such models have been described in more detail in other places; however, there is clear benefit from adopting such perspective and analyzing the use of equipment in the specific context of its use [17, 18].

## Notes

***Acknowledgments.*** The authors thank Stevi Gire, who helped to develop the scenarios.

***Financial support.*** This work was supported by the Centers for Disease Control and Prevention (grant number P50 CA098252).

***Supplement sponsorship.*** This article appears as part of the supplement “Personal Protective Equipment for Preventing Contact Transmission of Pathogens: Innovations from CDC’s Prevention Epicenters Program,” sponsored by the CDC’s Prevention Epicenters Program.

***Potential conflicts of interest.*** The authors: No reported conflicts of interest. All authors have submitted the ICMJE Form for Disclosure of Potential Conflicts of Interest. Conflicts that the editors consider relevant to the content of the manuscript have been disclosed.
